# PEP1 of *Arabis alpina* Is Encoded by Two Overlapping Genes That Contribute to Natural Genetic Variation in Perennial Flowering

**DOI:** 10.1371/journal.pgen.1003130

**Published:** 2012-12-20

**Authors:** Maria C. Albani, Loren Castaings, Stefan Wötzel, Julieta L. Mateos, Jörg Wunder, Renhou Wang, Mathieu Reymond, George Coupland

**Affiliations:** Max Planck Institute for Plant Breeding Research, Cologne, Germany; National University of Singapore and Temasek Life Sciences Laboratory, Singapore

## Abstract

Higher plants exhibit a variety of different life histories. Annual plants live for less than a year and after flowering produce seeds and senesce. By contrast perennials live for many years, dividing their life cycle into episodes of vegetative growth and flowering. Environmental cues control key check points in both life histories. Genes controlling responses to these cues exhibit natural genetic variation that has been studied most in short-lived annuals. We characterize natural genetic variation conferring differences in the perennial life cycle of *Arabis alpina*. Previously the accession Pajares was shown to flower after prolonged exposure to cold (vernalization) and only for a limited period before returning to vegetative growth. We describe five accessions of *A. alpina* that do not require vernalization to flower and flower continuously. Genetic complementation showed that these accessions carry mutant alleles at *PERPETUAL FLOWERING 1* (*PEP1*), which encodes a MADS box transcription factor orthologous to FLOWERING LOCUS C in the annual *Arabidopsis thaliana*. Each accession carries a different mutation at *PEP1*, suggesting that such variation has arisen independently many times. Characterization of these alleles demonstrated that in most accessions, including Pajares, the *PEP1* locus contains a tandem arrangement of a full length and a partial *PEP1* copy, which give rise to two full-length transcripts that are differentially expressed. This complexity contrasts with the single gene present in *A. thaliana* and might contribute to the more complex expression pattern of *PEP1* that is associated with the perennial life-cycle. Our work demonstrates that natural accessions of *A. alpina* exhibit distinct life histories conferred by differences in PEP1 activity, and that continuous flowering forms have arisen multiple times by inactivation of the floral repressor *PEP1*. Similar phenotypic variation is found in other herbaceous perennial species, and our results provide a paradigm for how characteristic perennial phenotypes might arise.

## Author Summary

Perennial plants live for many years and cycle between flowering and vegetative growth. These stages of the life cycle are often initiated by environmental conditions and occur seasonally. However, many herbaceous perennial species such as strawberry, rose, or *Arabis alpina* contain varieties that flower continuously irrespective of the seasons. Here we characterize this genetic variation in *A. alpina* and show that five continuously flowering accessions carry independent mutations in the *PERPETUAL FLOWERING 1* (*PEP1*) gene. These mutations impair the activity of the PEP1 floral repressor causing the plants to flower without requirement for winter cold and to flower continuously. This result has interesting parallels with strawberry and rose, where inactivation of a different floral repressor controlling response to day length gives rise to naturally occurring perpetual flowering forms. We also show that *PEP1* in *A. alpina* has a complex duplicated structure that gives rise to two overlapping transcripts. This arrangement differs from the simple structure of *PEP1* orthologues in related annual species, such as *FLC* of *Arabidopsis thaliana*, suggesting that duplication of *PEP1* might contribute to the complex transcriptional patterns associated with *PEP1* function in perennials. Our work provides insight into genetic variation contributing to the perennial life history of plants.

## Introduction

Key stages in the plant life cycle are regulated in response to environmental cues. Many genes controlling these responses show allelic variation within species allowing adaptation of individuals to their environment. Short-lived annual plants exhibit natural genetic variation for seasonal flowering responses that has been studied extensively in *Arabidopsis thaliana* [1], [2] and several crops [3]. The transition from vegetative to reproductive development in annuals marks the end of their life cycle. In contrast, perennials live for many years and undergo repeated cycles of vegetative growth and flowering. Most temperate perennial species flower seasonally and the phases of flowering and vegetative growth are clearly separated. Interestingly, in perennials the environmental cues that regulate floral initiation also have an impact on the duration of the flowering episode either by regulating the growth of flower buds [4] or the return to vegetative development [5], [6], [7]. Genetic variation also exists for these traits so that individuals of the same species flower either for a restricted period or for a more prolonged time [4], [8], [9]. In addition, differences in activity of flowering genes can contribute to the divergence in life history between plant species, including the distinction between annual and perennial life cycles [5], [10], [11], [12], [13], [14], [15]. Analysis of species within the Brassicaceae family provides an opportunity to extend the intensive knowledge of mechanisms of flowering-time control in the model annual species *A. thaliana* to closely related perennial species.

The evolution of adaptive traits that contribute to the annual or perennial life strategies can be relatively rapid and in a small number of examples has been associated with genetic alterations such as inversions, gene duplications or modifications in gene expression patterns [5], [10], [11], [14], [16], [17], [18], [19]. We showed previously that the MADS box transcription factor PEP1 regulates flowering and perennial specific traits in the Brassicaceae species *A. alpina* [5]. So far, flowering and the perennial growth habit of *A. alpina* have been characterized only in the accession Pajares (Paj), which was collected in the Cardillera Cantábrica mountain region of Spain [5]. This accession exhibits an obligate requirement for prolonged exposure to cold (vernalization) in order to flower. *PEP1* represses flowering before vernalization, so that *pep1-1* mutants identified after mutagenesis flower rapidly without vernalization [5]. In addition, *pep1-1* mutant plants flower for a longer period than wild-type plants linking vernalization requirement to the duration of a flowering episode.

*PEP1* is the orthologue of *A. thaliana FLOWERING LOCUS C* (*FLC*), which also encodes a floral repressor that delays flowering prior to vernalization. In *A. thaliana*, FLC delays flowering by repressing transcription of genes necessary for the switch to reproductive development [20], [21], [22]. This block on flowering is overcome by vernalization, which reduces *FLC* expression allowing flowering to proceed [23], [24]. *FLC* mRNA levels in *A. thaliana* are stably repressed after vernalization ensuring that all lateral branches that are formed after floral initiation also flower and produce seeds. *A. thaliana* plants then die, completing the annual life cycle. Stable *FLC* repression after flowering is therefore important in ensuring that all branches undergo flowering, maximizing seed production within the single year of the life cycle. The stable repression of *FLC* is associated with epigenetic changes at the locus, which involve trimethylation on lysine 27 of histone 3 (H3K27me3) at the *FLC* gene. The H3K27me3 mark spreads after vernalization at the *FLC* locus [25], [26] stably repressing its transcription.

Most natural genetic variation in *A. thaliana* for flowering time in response to vernalization is conferred by allelic variation at *FLC* or its upstream regulator *FRIGIDA* (*FRI*) [27], [28], [29], [30], [31], [32], [33]. FRI promotes *FLC* transcription, so that loss of FRI activity results in low *FLC* mRNA levels [23], [24]. Early flowering *A. thaliana* accessions that flower without vernalization are summer annuals. Most of these show low *FLC* mRNA levels either because they carry lesions in *FRI* or mutations at the *FLC* locus that prevent its upregulation by FRI [27], [28], [29], [31], [34], [35]. Early-flowering accessions with high levels of *FLC* mRNA have also been reported and carry mutations at the *FLC* locus [28], [36]. By contrast, all winter-annual accessions, which are late flowering and exhibit a strong vernalization requirement, express *FLC* mRNA at high levels [23], [24], [27], [29], [30]. Natural genetic variation among winter annual *A. thaliana* accessions has been described for the extent of vernalization period required to promote flowering and is also associated with allelic variation at *FLC* [37], [38].

In *A. alpina*, *PEP1* has a similar role to *FLC* conferring a response to vernalization, but in addition it contributes to perennial flowering traits not found in *A. thaliana*. *PEP1* mRNA levels in *A. alpina* are not stably repressed by vernalization, even after a saturating vernalization period that initiates flowering. Rather, *PEP1* mRNA levels rise again when plants experience warm temperatures after winter. Similar expression patterns of *FLC* orthologues have been described in another perennial Brassicaceae species, *Arabidopsis halleri* [19]. The rise in *PEP1* mRNA levels after vernalization blocks flowering of any shoots that have not already flowered, causing the plant to return to vegetative growth. The accumulation of trimethylation of lysine 27 of histone 3 (H3K27me3) increases at *PEP1* locus during vernalization and is associated with repression of transcription [25], [26]. However, contrary to *FLC* in *A. thaliana,* the H3K27me3 mark is not maintained after cold and this correlates with reactivation of *PEP1* transcription [5], [25], [26].

Here we identify *A. alpina* accessions that flower without vernalization and show that these are also naturally occurring perpetual flowering accessions. Five of these accessions were characterized at the genetic and molecular levels and in each case the natural phenotypic variation is caused by loss of function alleles at *PEP1.* These five accessions carry different lesions at the *PEP1* gene suggesting that they have arisen independently during evolution. Analysis of this variation also demonstrated that *A. alpina* contains a segmental partial duplication of *PEP1* that created two transcriptional starts sites and two overlapping transcripts, a more complex structure than is found at *FLC* in *A. thaliana*. We discuss this intra- and inter-species variation of *PEP1* structure and function in terms of evolution of life history traits in the Brassicaceae.

## Results

### *PEP1* contributes to variation in flowering behavior among *A. alpina* accessions

**Figure 1:**
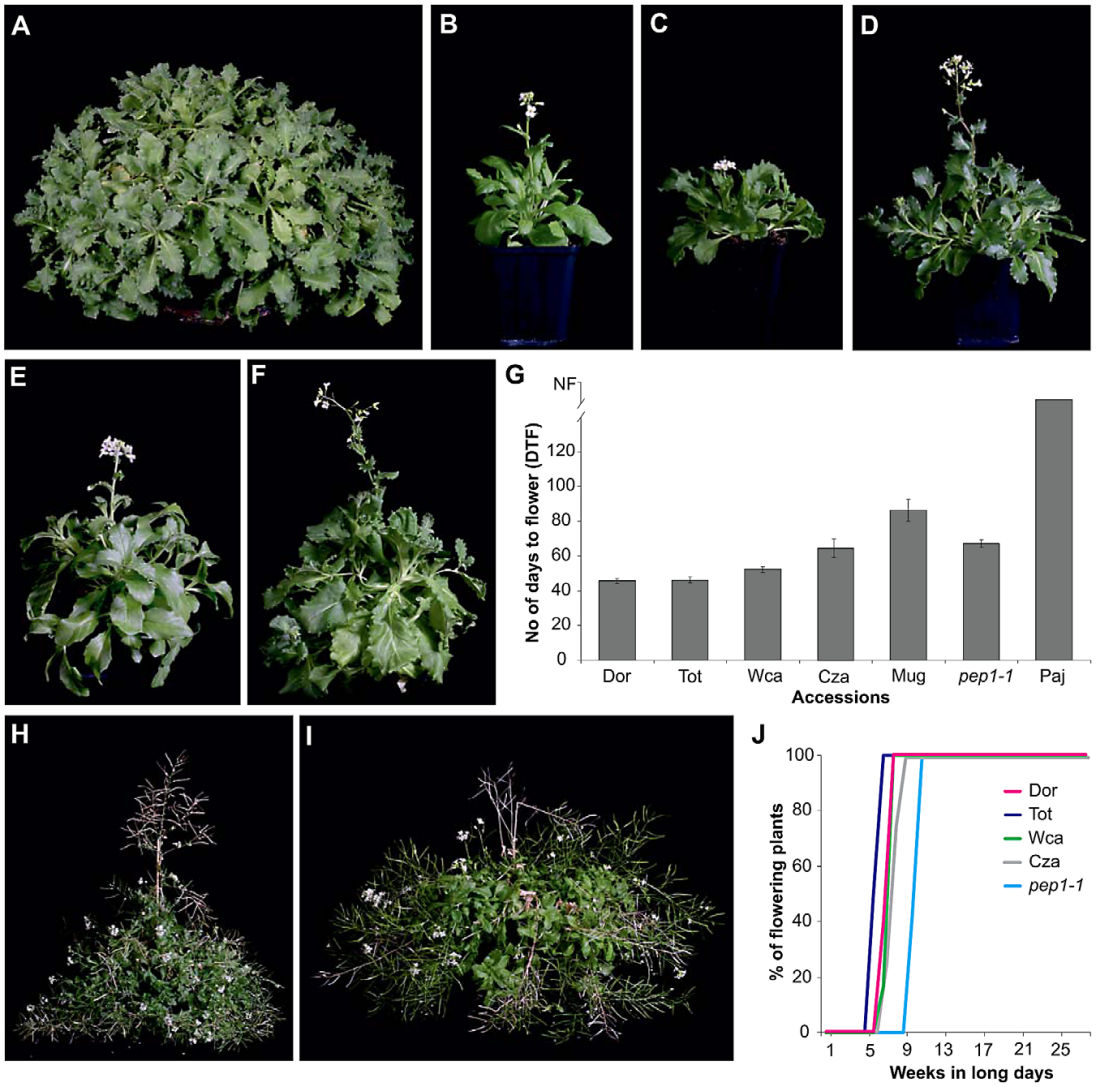
Flowering behavior of *A. alpina* accessions. (A) Accession Paj grown vegetatively for four years in long day glasshouse. Paj has an obligate requirement for vernalization to flower. (B)–(I) Non-vernalization requiring *A. alpina* accessions at flowering under long days. Accession Dor (B), Tot (C), Wca (D), Cza (E) and Mug (F). (G) Flowering times of non-vernalization requiring *A. alpina* accessions under long days (16 hours light) compared to *pep1-1* mutant and the accession Paj. Flowering time is measured as days to flower (DTF). *pep1-1* mutant (H) and the accession Dor (I) flower perpetually after 6 months in long days. (J) Duration of flowering in non-vernalization requiring *A. alpina* accessions.

The accession Paj carries an active allele of *PEP1* and has an obligate vernalization requirement to flower (Figure 1A; [5]). EMS-induced mutations in *PEP1* abolish the obligate vernalization requirement causing Paj to flower within 60 days in long days [5]. To test whether allelic differences at *PEP1* contribute to natural variation for perennial flowering traits, the flowering times of 24 accessions (Table S1) from diverse locations were assessed in long days. Five accessions that flowered without vernalization and within 3 months of germination were identified as candidates for carrying natural *pep1* mutant alleles. Four of these accessions, Dorfertal (Dor), Totes Gebirge (Tot), West Carpathians (Wca) and Czarna Gòra (Cza), flowered even earlier than the *pep1-1* mutant, whereas the accession Muggendorf (Mug) flowered slightly later than *pep1-1* (Figure 1B–1G, Figure S1). The *pep1-1* mutant plants flower perpetually indicating that *PEP1* also regulates the duration of flowering and return to vegetative development (Figure 1H; [5]). To test whether flowering without vernalization in the *A. alpina* accessions also correlates with perpetual flowering, the accessions Dor, Tot, Cza and Wca were grown under long days and their duration of flowering was compared to the *pep1-1* mutant. All accessions were still flowering 28 weeks after flower initiation, demonstrating that flowering without vernalization correlates with the perpetual flowering trait in natural *A. alpina* accessions (Figure 1H–1J).

**Figure 2:**
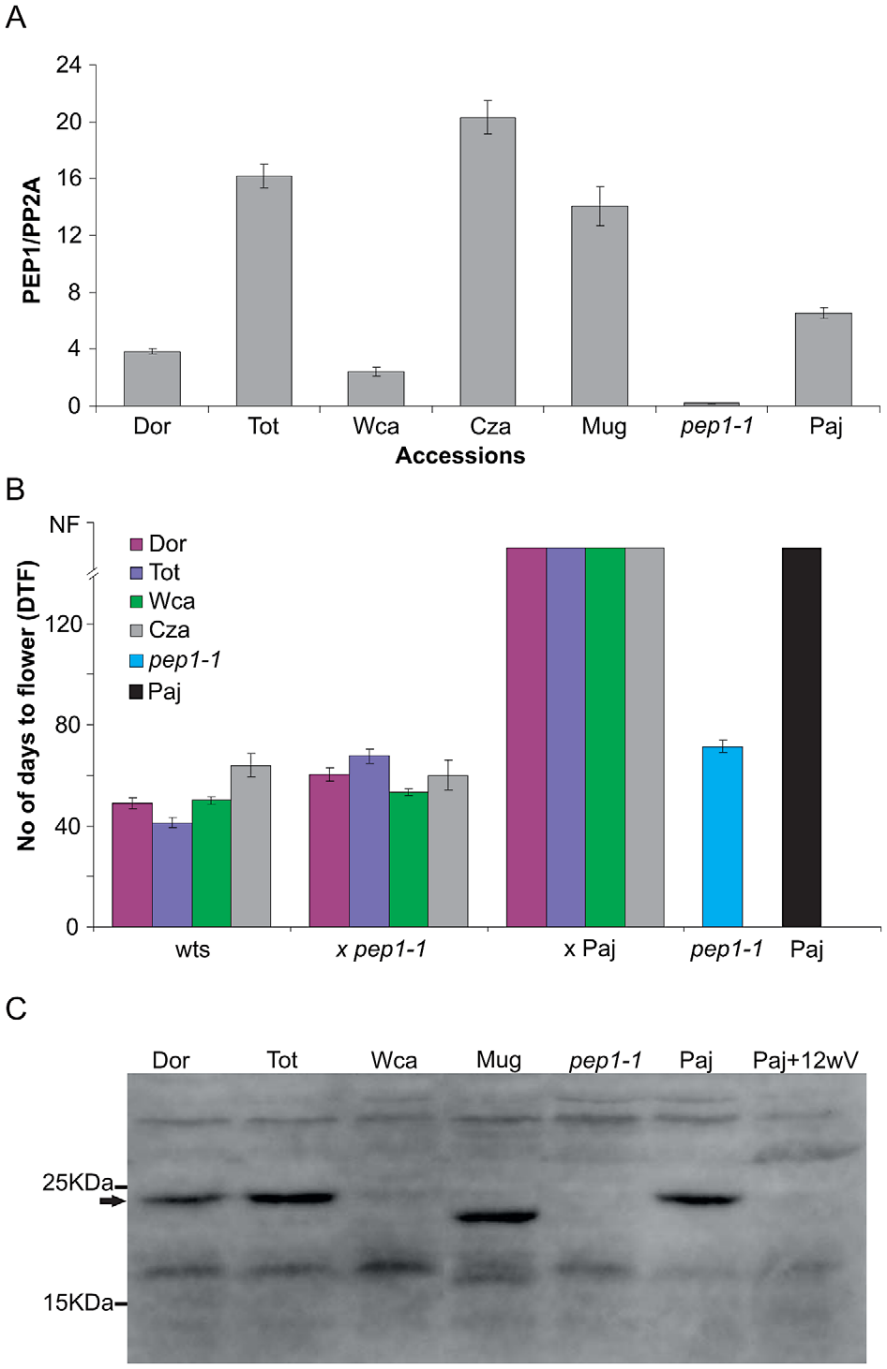
Non-vernalization requiring accessions do not rescue the early flowering phenotype of the *pep1-1* mutant. (A) *PEP1* mRNA levels in leaves of non-vernalization requiring accessions compared to Paj. (B) Flowering time of F1 hybrids resulted from crosses of non-vernalization requiring accession with *pep1-1* mutant and Paj in long days without vernalization. The *pep1-1* mutant and Paj were used as controls. Flowering time is measured as days to flower (DTF). (C) PEP1 accumulation in different accessions compared to the accession Paj before vernalization. *pep1-1* and Paj after 16 weeks in vernalization were used as negative controls. A cross reacting protein acts as a loading control.

In *A. thaliana FLC* mRNA levels often correlate with flowering time [23]. Therefore we compared the *PEP1* mRNA levels of the early-flowering accessions that do not require vernalization to flower with those of the accession Paj that has an obligate vernalization requirement. Most accessions exhibited *PEP1* mRNA levels at least as high as those found in the accession Paj suggesting that flowering without vernalization did not obviously correlate with reduced *PEP1* mRNA (Figure 2A). However, the accessions Dor and Wca had lower *PEP1* mRNA levels than Paj. Therefore reduced *PEP1* mRNA levels in these accessions might explain their flowering phenotypes, but this cannot be the basis of early flowering in the accessions Mug, Cza and Tot.

These non-vernalization requiring accessions were then crossed to *pep1-1* to test whether they carry a *PEP1* allele that can complement the *pep1-1* mutation. As control, these accessions were also crossed to Paj and the flowering times of F1 plants were measured (Figure 2B, Figure S2). All F1 plants derived from the crosses to the *pep1-1* mutant flowered without vernalization, suggesting that these accessions carry non-functional *PEP1* alleles that cannot complement the *pep1-1* mutation (Figure 2B). By contrast all F1 plants derived from the crosses to Paj did not flower for several months when grown in long days, indicating that the functional allele of *PEP1* from Paj is sufficient to delay flowering of the F1 plants. Taken together these genetic experiments suggest that the non-vernalization requiring accessions carry inactive alleles of *PEP1*.

To test whether they carry mutations in the *PEP1* open reading frame that might account for the non-functional *PEP1* alleles we sequenced *PEP1* cDNAs from each accession (Table 1, Table S2). Primers that anneal to the -5′ and -3′ UTRs were used to amplify 814 bp of coding sequence from apices prior to vernalization. Amplification products from each accession were then cloned in *Escherichia coli* and several clones were sequenced. *PEP1* mRNA from Paj was shown to be alternatively spliced generating forms that retained introns or that had lost parts of or whole exons [5]. Most of the sequenced clones from the accessions contained the full length ORF suggesting that this is the predominant transcript although splicing variants were also detected (Table 1, Table S2). The full-length cDNAs isolated from the Dor, Tot, Cza and Mug accessions contained polymorphisms that caused amino acid substitutions or deletions compared to the Paj allele (Table 1), suggesting that these polymorphisms might be the basis of loss of PEP1 activity and therefore the altered flowering behavior. In the accession Wca the predominant cDNA sequence did not contain any amino acid substitutions or deletions when compared to Paj and thus the reduced PEP1 activity is probably due to the low mRNA level described above.

**Table 1:** Comparison of *PEP1* coding sequences of five early-flowering *A. alpina* accessions and the obligate-vernalization requiring Paj accession.

	Clones	5′UTR	Exon 1		Exon 3				Exon 4	
**nucl.**			100	180	329	336–362	377–379		433	441
**a.a**			6E/K		83L/R	85–93-SLLHGQDLQ	99-Y	100G/C	118V/I	
**Paj**			G	T	T				G	C
**Dor**	262	+248	AG							
**Tot**	24								A	T
**Wca**	11									
**Cza**	17				G		−3			
**Mug**	10			A		−27				

Multiple cDNAs were analyzed for the accessions Dor, Tot, Wca, Cza and Mug and the numbers of cDNA sequences recovered is shown in the “Clones” column. The full-length *PEP1* cDNA sequence of the vernalization-requiring accession Paj is used as a reference (row highlighted in grey). Nucleotide polymorphisms compared to Paj *PEP1* cDNA sequence obtained for each accession are presented. Nucleotide (nucl.) position and aminoacid (a.a.) changes compared to Paj mentioned in rows above the grey row. For most accessions, different *PEP1* spicing forms were also recovered and are presented in Table S2.

Apart from the splicing variants the polymorphisms found within the *PEP1* cDNA sequences were different for each of the non-vernalization requiring accessions (Table 1). From the Dor accession cDNAs containing polymorphisms in exon 1 were amplified. Most cDNAs contained a G to A base substitution in exon 1 that is predicted to cause an amino acid substitution in the highly conserved MADS box domain responsible for DNA binding whereas some sequences were identical to that of Paj (Figure S3). The origin of these different cDNA sequences is discussed below. The *PEP1* cDNA isolated from accession Tot contained two base substitutions in exon 4, which encodes the K box, and one was predicted to cause a valine (Val) to isoleucine (Ile) amino acid substitution compared to Paj. The accession Cza contained a base substitution and a 3 bp deletion in exon 3. Compared to Paj the base substitution is predicted to cause a leucine (Leu) to arginine (Arg) change, whilst the deletion is expected to cause loss of glycine (Gly) and tyrosine (Tyr) residues and their replacement by a cysteine (Cys) amino acid. Finally, the accession Mug contained a synonymous base substitution in exon 1 and a 27 bp deletion in exon 3, which resulted in a predicted loss of 9 amino acids in the K box domain of the protein.

To test if PEP1 protein accumulation correlated with flowering behavior in the accessions an antibody was raised against the protein (Methods). PEP1 protein was then tested using Western blots of apical samples of the non-vernalization requiring accessions, Paj and *pep1-1* before vernalization, and vernalized Paj plants (Figure 2C). PEP1 protein was detected in Paj before vernalization but not after 12 weeks vernalization and was absent from the *pep1-1* mutant. In the accession Wca PEP1 was not detected, which correlates with low *PEP1* mRNA levels compared to the accession Paj (Figure 2A, 2C). PEP1 protein in the accession Mug was smaller, consistent with the 27 bp in frame deletion in exon 3 in this accession (Figure 2C, Table 1). The accumulation of PEP1 in the accessions Dor and Tot was similar to the accession Paj before vernalization, although as described above the forms of PEP1 in Dor and Tot are predicted to contain amino acid changes compared to Paj.

Taken together these data indicate that some non-vernalization requiring accessions carry *PEP1* alleles with polymorphisms in the protein coding sequence that likely impair protein function, whilst Wca shows reduced PEP1 accumulation. The genetic and molecular analyses of these accessions suggest that these natural alleles contribute to loss of vernalization requirement and perpetual flowering by reducing PEP1 protein level or activity.

### Sequence variation in *PEP1* cDNAs of the Dor accession reveals the complex structure of the *PEP1* locus

**Figure 3:**
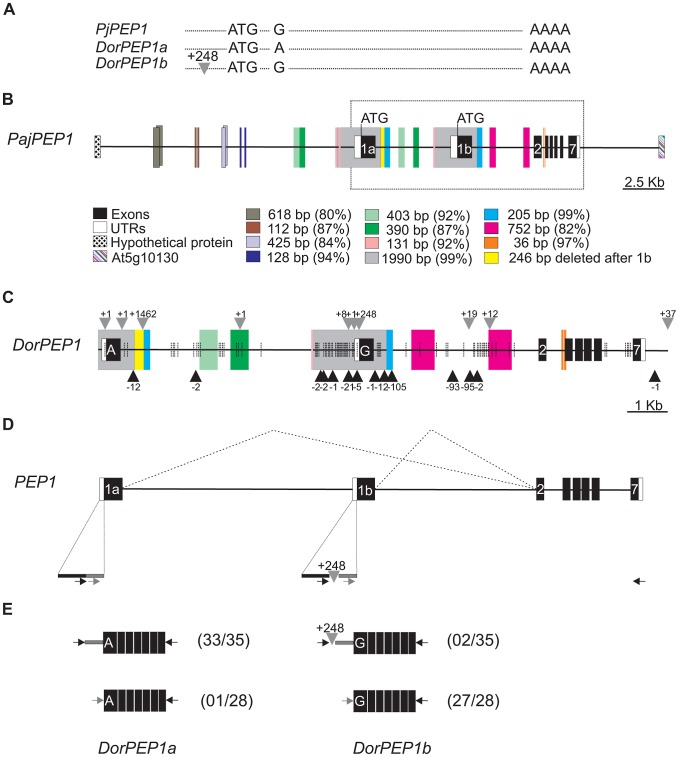
Analysis of sequence variation in *PEP1* cDNA and at the genomic locus of Dor accession demonstrates a complex structure for the *PEP1* gene. (A) *PEP1* cDNAs in Dor is a mixture of transcripts that contain a G to A substitution in exon 1 compared to Paj or have a similar sequence to accession Paj but have an insertion of 248 bp in the 5′ UTR. (B) Sequence of the *PEP1* genomic locus in the accession Paj shows that the locus is highly duplicated. Exons are indicated with black boxes, UTRs with white boxes and solid lines the inter- and intra-genic regions. Upstream and downstream genes of *PEP1* are 35 kb apart. Colored boxes indicate relative positions of the duplicated regions. Overlapping boxes indicate overlapping homologous sequences. Numbers besides duplicated boxes show the length of the duplicated fragment and percentage of homology. Duplicated exon 1 copies are indicated as 1a and 1b. Dotted box shows the *PEP1* locus region sequenced in the accession Dor. (C) Sequence of the *PEP1* genomic locus in the Dor accession reveals that G to A base substitution is in exon 1a. Grey arrows indicate insertions, black arrows indicate deletions and vertical dotted lines indicate SNPs relative to Paj *PEP1* locus. The 248 bp insertion upstream in the 5′ UTR is upstream of exon 1b. Colored boxes indicate relative positions of duplicated regions. (D) Structure of the *PEP1* locus and predicted splicing events (E) *PEP1* transcripts in the accession Dor detected with two different primers in the 5′ UTR using the same reverse primer in the 3′ UTR. Black and grey arrows indicate the position of two different primers in the 5′ UTR relative to the 248 bp insertion. When primer PEP1_5UTRF1 (black) was used most clones contained the G to A substitution in the exon 1. A few clones that did not contain the G to A base substitution also contained a 248 bp insertion in the 5′ UTR. When primer PEP1_5UTRF2 was used most clones did not contain G to A base substitution.

The *PEP1* cDNAs isolated from the accession Dor varied in the sequence of exon 1, being either identical to Paj or carrying a non-synonymous change (Table 1, Figure 3A). These different cDNAs could not be explained by Dor being heterozygous at *PEP1*, because genetic experiments demonstrated that it carries the same allele on both chromosomes. Most Dor *PEP1* cDNAs contained the G to A base substitution compared to Paj at the beginning of the first exon (Table 1). The other set of cDNAs encoding an ORF identical to the accession Paj also contained a 248 bp insertion in the 5′ UTR region, which was absent from Paj cDNA and absent from the predominant transcript carrying the G to A substitution (Table 1). The presence of both *PEP1* cDNAs in Dor was verified using a different primer in the 5′ UTR and the same reverse 3′ UTR primer. Again both types of cDNA were detected, but surprisingly their ratio was altered so that most clones contained sequences similar to accession Paj and only one contained the G to A base substitution (Figure 3E).

The *PEP1* locus in the accession Paj is partially duplicated and is located in a region of the *A. alpina* genome that shows conserved synteny with *A. thaliana* on chromosome 5 where *FLC* is located. In *A. alpina* Paj the distance between the orthologues of the genes that flank *FLC* in *A. thaliana* (At5g10130 and At5g10150) is increased [5], and this region includes several tandemly duplicated sequences that include parts of *PEP1* (Figure 3B). The longest duplicated segment is approximately 2 kb and contains a copy of exon 1 of *PEP1*, as well as parts of the *PEP1* promoter and intron 1. The tandem copies of exon 1, named 1a and 1b are located 6.3 kb apart (Figure 3B). To determine whether the two types of *PEP1* cDNA identified in the Dor accession are encoded by these different copies of exon 1, the *PEP1* locus was sequenced in the accession Dor using a BAC containing the entire *PEP1* locus. The contiguous region from exon 1b to exon 7 showed a similar structure to *PEP1* from Paj. Furthermore, exon 1b showed the same sequence as Paj but contained the 248 bp insertion in the 5′ UTR found in some of the Dor cDNAs described above (Figure 3A, Table 1). Therefore those Dor *PEP1* cDNAs encoding the same ORF as Paj and the insertion in the 5′ UTR were encoded by the contiguous region from exon 1b to exon 7, but the cDNAs encoding the non-synonymous polymorphism in exon 1 were not derived from this region. A 10 kb region upstream of *PEP1* exon 1b in accession Dor was then amplified, cloned and sequenced. A second copy of *PEP1* exon 1, which corresponded to exon 1a in Paj, was present in this upstream region, and this contained the G to A base substitution in the first exon which was detected in the second group of *PEP1* cDNAs found in the Dor accession (Figure 3C). This result indicated that exon 1a is also used to produce a full length *PEP1* transcript and both exon 1a and exon 1b are spliced to the same copy of exon 2 to produce two transcripts that share exons 2–7 (Figure 3D). Which transcript is amplified preferentially from Dor accession depends on the primer in the 5′ UTR used which is probably influenced by the insertion in the 5′ UTR of the exon 1b (Figure 3E).

### The *PEP1* locus contains tandem duplications in other *A. alpina* accessions

**Figure 4:**
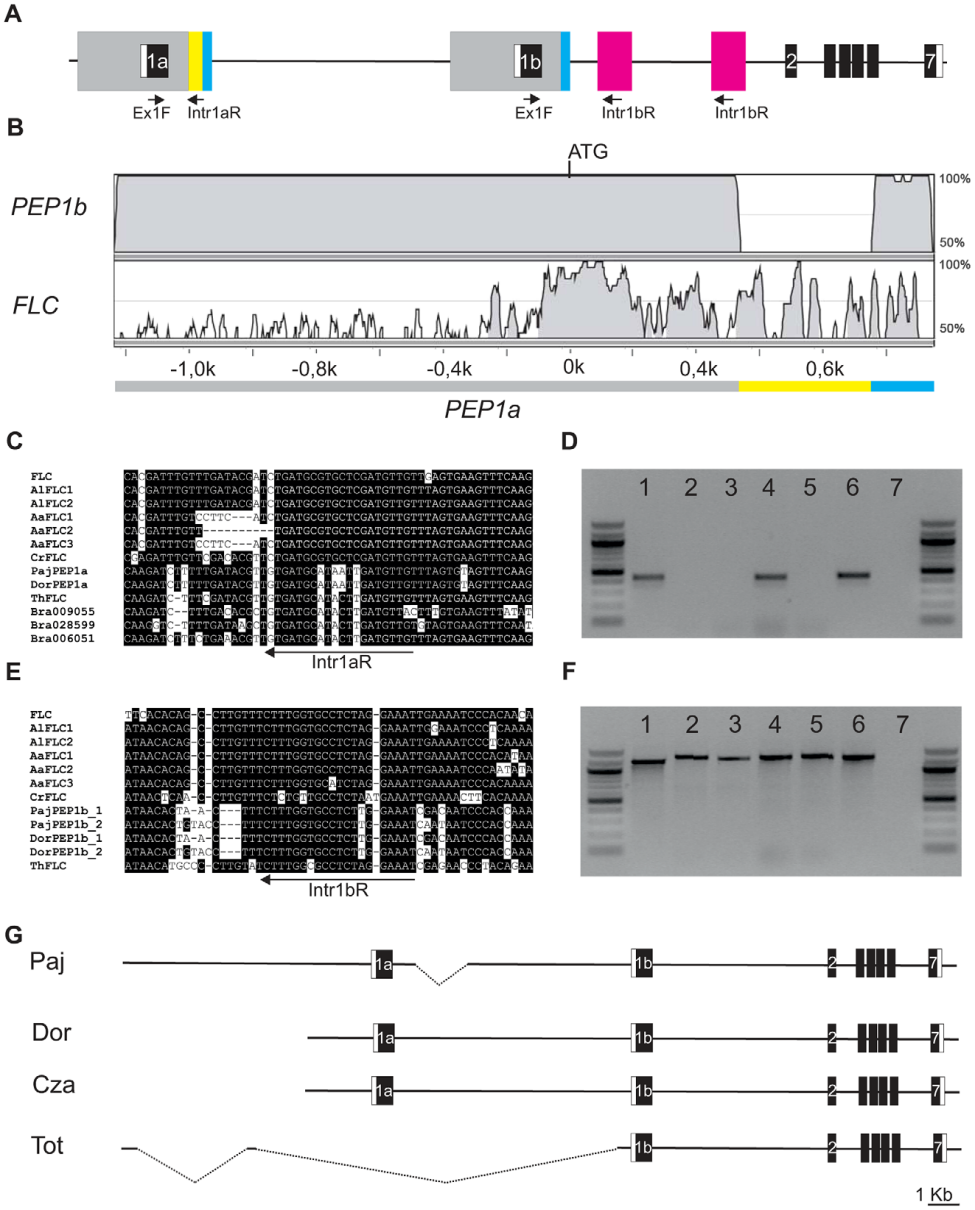
*PEP1* locus is tandemly duplicated in several *A. alpina* accessions. (A) Structure of the *PEP1* locus and the position of exon 1a and exon 1b specific primers. (B) Sequence comparisons of *FLC* and tandem duplicated copies 1a and 1b in accession Paj (grey to blue boxes in Figure 3A). Vista plot using Calc. window 25, Min cons width 25 and Cons identity 70%. (C) Alignment using part of the 416 bp sequence (yellow box) specific for exon 1a from different *FLC* homologues. Intr1aR primer was designed in a consensus sequence. (D) PCR test using *PEP1a* specific primers (Ex1F and Intr1aR) in different accessions. Template used is Dor (1), Tot (2), Wca (3), Cza (4), Mug (5) Paj (6) and water control (7). (E) Alignment using part of the intron sequence downstream of *PEP1b.* Intr1bR primer was designed in a duplicated region (pink box in Figure 3C, 3B, Figure 4A) conserved in other *FLC* homologues. (F) PCR test using *PEP1b* specific primers (Ex1F and Intr1bR) in different accessions. Template used is Dor (1), Tot (2), Wca (3), Cza (4), Mug (5) Paj (6) and water control (7). (G) *PEP1* structure of the accessions Paj, Dor, Cza and Tot obtained by sequencing the *PEP1* locus.

The duplicated regions flanking *PEP1* exons 1a and 1b in the accession Paj (grey to blue boxes in Figure 3B) are highly similar except for a 246 bp sequence after exon 1a that has been deleted from exon 1b region (yellow rectangle in Figure 3B, Figure 4A and 4B). This 246 bp deletion after exon 1b also exists in the accession Dor, indicating that it occurred before the divergence of the accessions Paj and Dor (Figure 3C). Moreover, this 246 bp sequence in Paj *PEP1a* shows homology with the first intron of *FLC* in *A. thaliana*, suggesting that in this respect *PEP1* exon 1a is more related to *FLC* than is *PEP1* exon 1b (Figure 4B).

To test if other *A. alpina* accessions also contained the tandem duplication at the *PEP1* locus specific primers were designed for *PEP1a* and *PEP1b* copies annealing to the 246 bp segment specific for exon 1a or to a conserved sequence in the first intron after exon 1b (Figure 4C, Figure 4E). These primers were used in combination with a common forward primer in the MADS box region that annealed to both *PEP1a* and *PEP1b* (Figure 4A, Figure 4C, Figure 4E). The *PEP1b* specific primers amplified a fragment in all accessions, indicating that *PEP1b* is likely conserved among these accessions (Figure 4F). However, these fragments varied slightly in size, consistent with the deletion present in the accession Dor (described below) compared to Paj. By contrast the *PEP1a* specific primers amplified a fragment in the accessions Dor, Cza and Paj, but not in Tot, Wca or Mug (Figure 4D). This result indicates that the *PEP1b* region is more conserved than *PEP1a* among *A. alpina* accessions.

The genomic *PEP1* loci from the accessions Cza and Tot were then sequenced to analyze their structure in detail. The *PEP1* allele in the accession Cza exhibited a similar structure to the accessions Dor and Paj, containing both exon 1a and exon 1b (Figure 4G). No fragment corresponding to exon 1a was amplified from accession Tot. To provide genomic information for this region, Illumina sequencing was performed on DNA extracted from Tot. The sequence reads were assembled and searched for homology to *PEP1* using BLAST. This sequence information was then used to design locus specific primers to test the structure of *PEP1* in Tot and to provide fragments for Sanger sequencing. This analysis demonstrated that the Tot allele contained only exon 1b, and had suffered a deletion that includes exon 1a and the intergenic region between exon1a and 1b. Therefore loss of *PEP1* function in this allele correlates with absence of the *PEP1a* region as well as the non-synonymous substitution in exon 4.

### *PEP1a* and *PEP1b* transcripts are unstably and differentially expressed after vernalization in the accession Dor

**Figure 5:**
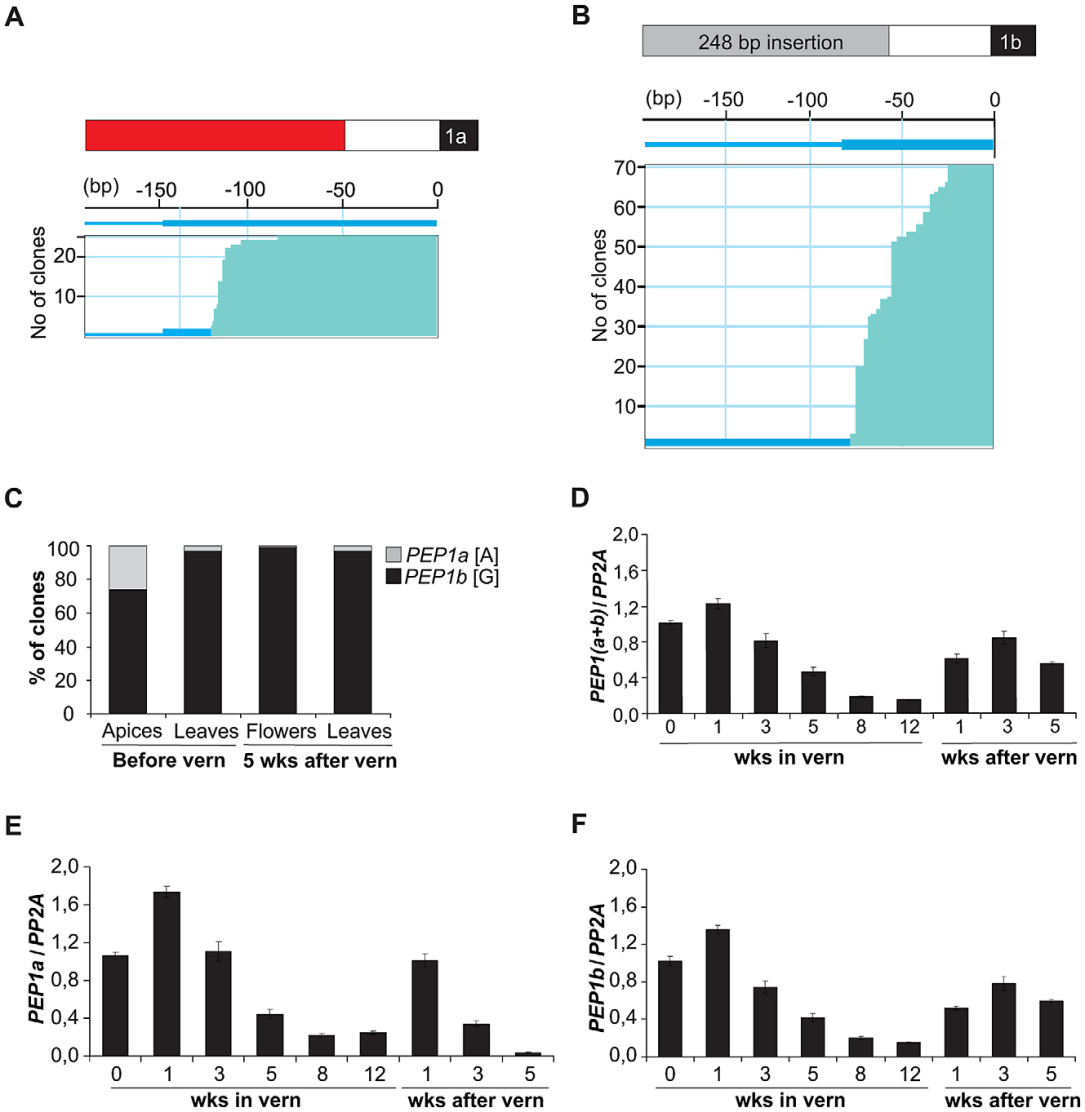
*PEP1a* and *PEP1b* genes in the accession Dor are independently transcribed and have different transcriptional start sites. (A,B) Number of clones containing G to A polymorphism on exon 1 (A) or not (B) after 5′ RACE using apices from Dor plants growing for 3 weeks in long days. Schematic representation of exon1 and 5′ UTR regions (top), exon1a and exon 1b (black boxes), sequence present in 5′ UTR upstream of both exon 1a and 1b (white box), sequence specific to 5′ UTR upstream of exon 1a (red box), sequence specific to 5′ UTR upstream exon 1b on the 248 bp insertion (grey box). Horizontal lines represent individual clones. Numbers on the top represent bp upstream of ATGs. (B,C) Percentage of clones with the A or G polymorphism after 5′ RACE in apices and leaves before (3 week long days) and after vernalization (5 weeks in long days after 12 weeks vernalization). (D)–(F) *PEP1* mRNA levels on 3 week old Dor plants, vernalized for 12 weeks and subsequently grown for several week in long days. (D) *PEP1* (a+b) expression, primers used similar as in [5] to detect both transcripts (E) *PEP1a* expression, primers used to detect only *PEP1a* transcripts. (F) *PEP1b* expression, primers used to detect only *PEP1b* transcripts.

The sequences upstream of *PEP1* exon 1a and exon 1b are highly diverged (Figure 3B), suggesting that the two *PEP1* genes might be expressed from different promoters. To test whether the *PEP1* transcripts encoding exon 1a and exon 1b use two different transcription start sites 5′ RACE was performed on the accession Dor in which the two copies of exon 1 can be differentiated. This analysis showed that exon 1a and exon 1b have distinct transcription start sites located 65–186 bp upstream of the ATG for exon 1a and 61–190 bp upstream of the ATG of exon 1b (Figure 5A, 5B).

To determine whether these different transcripts are differentially expressed 5′ RACE was performed in the accession Dor in apices and leaves before and after vernalization (Figure 5C). *PEP1a* polymorphism in the accession Dor was mainly found in transcripts from vegetative apices before vernalization representing 26% of the total *PEP1* mRNA pool. In leaves before and after vernalization Dor *PEP1a* was detected only in 3% of the transcripts representing only a small proportion of the total *PEP1* transcripts whereas was virtually undetectable in flowers five weeks after vernalization. By contrast Dor *PEP1b* was strongly expressed in apices, flowers and leaves in all stages.

To compare *PEP1a* and *PEP1b* transcript levels more thoroughly after vernalization, their expression patterns were followed through a time course in 3 week old Dor seedlings vernalized for 12 weeks and then returned to normal temperatures for an additional 1, 3 or 5 weeks. Both transcripts were repressed during vernalization and increased in expression after vernalization (Figure 5D, 5E, 5F). Dor *PEP1b* mRNA stayed at high levels for several weeks after vernalization, whereas Dor *PEP1a* mRNA levels fell again after 1 week in warm conditions.

These experiments indicate that the *PEP1a* and *PEP1b* genes are independently transcribed from different transcription start sites. Both transcripts show very similar patterns of transcriptional regulation before, during and immediately after vernalization, but *PEP1b* expression persists more strongly in flowers and leaves later after vernalization than *PEP1a* transcript.

## Discussion

*A. alpina* is an alpine perennial and the only previously characterized accession, Paj, flowers for a limited period in the spring after prolonged exposure to winter chilling temperatures. Flowering and perennial specific traits were studied in detail in this Spanish accession and shown to be under the control of *PEP1*, which is the orthologue of the *A. thaliana* gene *FLC* [5]. Here, we identified several *A. alpina* accessions that carry inactive *PEP1* alleles and hence do not require vernalization to flower. These accessions also flower perpetually similar to *pep1-1* mutant plants [5]. Therefore, among *A. alpina* accessions allelic variation at *PEP1* contributes to phenotypic differences in the duration of flowering as well as to differences in vernalization response. This is in contrast to loss of *FLC* function in *A. thaliana* accessions which is not associated with perpetual flowering due to its annual, monocarpic life cycle. The inactive alleles of *PEP1* are all different indicating that this phenotypic divergence has occurred many times in *A. alpina*. We found that the *PEP1* locus is partially duplicated including two copies of the first exon as well as parts of the proximal promoter and intron 1. These sequences are arranged as a tandem duplication creating two transcriptional start sites that generate two overlapping transcripts that are differentially expressed. This increased complexity at the *PEP1* locus might contribute to the contrasting transcriptional regulation of *PEP1* and *FLC* observed in perennial *A. alpina* and annual *A. thaliana* respectively.

### Intra-specific variation for seasonal flowering in herbaceous perennials

In *A. alpina* the correlation between perpetual flowering and the lack of vernalization requirement for floral induction was demonstrated by characterizing *pep1-1* which is an EMS induced mutant derived from mutagenesis of the Paj accession. Here we show that natural *A. alpina* accessions which do not require vernalization to initiate flowering also flower perpetually. This suggests that in nature *A. alpina* exhibits two life histories, a vernalization-requiring form that flowers for a limited period and a non-vernalization-requiring form that flowers continuously. Similar variation in the duration of flowering season also occurs in other herbaceous perennials such as strawberry and rose [8], [9], [39]. Cultivated strawberries and their wild relatives exhibit two distinct flowering habits and are classified either as seasonal-flowering (june bearing) producing one harvest per year or as perpetual-flowering (ever bearing) that produce two harvests per year [8], [39]. Interestingly, perpetual-flowering types in these Rosaceae species show different environmental requirements for flower induction compared to seasonal-flowering ones. Strawberries that follow the seasonal-flowering habit require short days and low temperatures to induce flowering, whereas perpetual-flowering strawberries have lost photoperiodic and temperature control of flowering or become long-day plants [7], [40], [41]. These types differ from *A. alpina* where accessions that flower perpetually because they lost vernalization requirement to flower. Interestingly, continuous flowering is also associated with subalpine forests compared to lowland forests [42] and the wild strawberry (*Fragaria vesca*) which follows the perpetual flowering habit is also called alpine strawberry because it is thought to have originated in the alps [43].

The duration of flowering season in *F. vesca* is controlled by a single gene, the *SEASONAL FLOWERING LOCUS* (*SFL*) [44]. Recently, sequence variation in the rose and *F. vesca* homologues of the *A. thaliana TERMINAL FLOWER 1* (*TFL1*) gene was shown to correlate with differences in the duration of flowering observed in seasonal and perpetual flowering types [6], [8], [45]. *F. vesca* accessions that flower perpetually carry a deletion in *F. vesca TFL1* (*FvTFL1*) that causes a premature stop codon whereas perpetual flowering in rose correlates with the presence of a retrotransposon in the second intron of the rose *TFL1* homologue (*KSN*) [6], [45]. *TFL1* encodes a protein related to phosphatidyl ethanolamine binding proteins and is a floral repressor that is specifically expressed at the shoot apical meristem [46], [47], [48], [49]. Transgenic experiments in *F. vesca* demonstrated that *FvTFL1* does not only regulate the duration of the flowering episode but also the induction of flowering in response to photoperiod, with short days in the autumn downregulating *FvTFL1* mRNA levels to initiate flowering [6]. In *A. alpina* reduction of *TFL1* function does not affect the duration of flowering season but rather causes plants to flower in response to vernalization earlier in their life cycle [10]. Thus although it does not define the duration of a flowering episode as it does in Rosaceae species, *TFL1* retains an important function in the perennial cycle of *A. alpina*. By contrast our work described here demonstrates that natural phenotypic variation between seasonal and perpetual forms of *A. alpina* is caused by loss of function alleles of *PEP1*. Therefore, genetic variation at genes encoding distinct classes of floral repressors appears to be responsible for differences in the duration of the flowering season among accessions of perennial species from different plant families. Recruitment of different flowering repressors to this function presumably explains how seasonal flowering phenotypes similar to those observed in *A. alpina* Pajares can occur in perennial species that do not contain *FLC* homologues such as *PEP1*.

Allelism tests indicated that all of the analyzed *A. alpina* accessions that flower perpetually and without vernalization carry inactive *PEP1* alleles. The perpetual flowering accessions carry independent mutations at *PEP1* that either cause amino acid substitutions, alter the structure of the locus or prevent protein accumulation and demonstrate that PEP1 activity was lost independently multiple times. In *A. thaliana* most natural phenotypic variation for flowering in response to vernalization can be explained by allelic variation at *FRI* [28], [29], [31], [34], [36]. Most summer annual accessions that flower without vernalization carry lesions at *FRI* and therefore flower early because they do not accumulate *FLC* mRNA to high levels [27], [29], [31]. Similarly, in *Arabidopsis lyrata* two alleles at the *FRI* orthologue were identified and were suggested to be associated with flowering time differences, although they did not appear to explain large differences in flowering time between populations [50]. *FRI*-independent variation in vernalization requirement also occurs among *A. thaliana* summer annual accessions and largely correlates with allelic variation at *FLC.* However, loss of function *FLC* alleles occur to a lesser extent than those in *FRI* [28], [35], [37], [38]. Some early-flowering summer annual accessions carry active *FRI* alleles but still show reduced *FLC* mRNA levels, which in some cases is associated with insertions in the first intron of *FLC* [34], [35]. Other accessions that express *FLC* mRNA at high levels carry mutations within the FLC protein coding sequence impairing its activity [28], [31], [36]. Our results provide no evidence for natural variation at *A. alpina FRI* playing a role in perennial flowering or vernalization requirement. Instead all early flowering *A. alpina* accessions tested carried mutations at the *FLC* orthologue *PEP1,* suggesting that natural variation at *PEP1* is the major source of variation in flowering response to vernalization and seasonal perennial flowering. Even in the accession Wca where PEP1 accumulation was low, consistent with reduced FRI activity, genetic complementation analysis demonstrated that the early flowering of this accession was due to a mutation at *PEP1*, similar to *A. thaliana* accessions that carry weak *FLC* alleles [34], [35].

In agreement with our data, QTLs affecting flowering time in Brassica crop species co-segregate with regions containing *FLC* homologues [51], [52], [53], [54], [55], [56], although there is also evidence for variation at *FRI* [57]. Studies on selected paralogue*s* in *Brassica. rapa, Brassica napus* and *Brassica oleraceae* support the idea that some *FLC* genes in these species might function in a similar way to *FLC* in *A. thaliana* and introduction of some of them, such as *BrFLC1-3* and *BnFLC1-5* into *A. thaliana* delayed flowering [58], [59]. Moreover, mRNA levels of some *FLC* paralogues correlate with flowering and vernalization requirement [56], [59].

Natural loss of function alleles of *PEP1* have arisen independently in several of the *A. alpina* populations analyzed. This might indicate that flowering without vernalization and/or perpetual flowering provide a selective advantage in some environments, perhaps because they allow production of more seeds over a longer growing period. Alternatively, *PEP1* loss of function alleles might provide no selective advantage but occur as neutral variation when selection on *PEP1* is relaxed at certain altitudes or in particular habitats. Understanding the evolutionary forces determining the prevalence of active and inactive *PEP1* alleles will require a future more detailed ecological analysis.

### Structure of the *PEP1* locus

*PEP1* in *A. alpina* has an unexpectedly complex structure including a partial tandem duplication giving rise to two overlapping mRNAs. The duplicated tandem segments include the first exon and adjacent sequences. Both copies of exon 1 (exon 1a and exon 1b) are spliced to the same copy of exon 2 and in the accession Paj are predicted to encode identical proteins. Alternative splicing of the single *FLC* copy in *A. thaliana* has also been described [60]. Most *A. alpina* accessions that carry inactive *PEP1* alleles have mutations in exons 2–7 that contribute to both proteins, and therefore the mutations inactivate both forms of the gene. Related overlapping plant gene configurations have been reported to form tissue specific isoforms of proteins based on the use of alternative promoters and/or alternative splicing of exons [61], [62], [63], [64], [65]. Studies in tropomyosins, for example, suggested that intragenic duplication events generated new exons that are combined in different ways utilizing alternative promoters, translational initiation sites or polyadenylation sites to produce tissue specific protein isoforms [62], [63], [66], [67], [68]. Nevertheless, in the accession Paj the two overlapping *PEP1* genes are predicted to encode identical proteins and therefore this structure has not evolved to encode different protein isoforms. However, the sequences upstream of each copy of exon 1 share little homology suggesting that the two overlapping copies are expressed from different promoters, and consistent with this interpretation they have independent transcription start sites. Furthermore, the expression patterns of the two *PEP1* transcripts are different suggesting that this complex structure might confer PEP1 activity in a broader range of tissues or developmental stages. In *A. thaliana* FLC functions to regulate flowering in leaves and apices [20] and other MADS box transcription factors have been shown to contribute to different transcriptional complexes at various stages in development [69]. A broader expression pattern of *PEP1* might allow it to contribute to a wider range of regulatory pathways.

Tandem duplications of *FLC* paralogues are found in *A. thaliana* and have been described for *FLC* orthologues in other members of the *Arabidopsis* genus [70]. A cluster of four genes encoding MADS-box transcription factors related to *FLC* occurs in *A. thaliana*. These genes, *MADS AFFECTING FLOWERING 2* (*MAF2*) to *MAF5*, delay flowering time but to a markedly lesser extent than *FLC* [71]. The tandem duplication of these four genes spans around 24 kb and allelic variation occurs among *A. thaliana* accessions, including a fusion of *MAF2* and *MAF3* to generate a chimeric gene [72]. The partial gene duplication of *PEP1* found in *A. alpina* might have arisen from such a rearrangement within an ancestral structure that includes a tandem array of full-length genes. Interestingly in *A. lyrata* and *Arabidopsis arenosa* tandem arrays of full-length *FLC* orthologues have been described in detail [70] and in *B. oleracea* a tandem duplication of *BoFLC1* was reported [73], suggesting that the single copy of *FLC* present in *A. thaliana* might be derived during evolution from an ancestral *FLC* locus that contained two or three tandem copies. *A. lyrata* harbours two full-length *FLC* genes whereas *A. arenosa* contains two copies and a partial copy. These were proposed to have arisen within the *Arabidopsis* genus and therefore would represent an independent duplication event to the one we describe in *A. alpina*. Nevertheless, as *A. arenosa*, *A. lyrata* and *A. alpina* are all perennials, and *PEP1*/*FLC* shows a more complex expression pattern in perennials than annuals, tandem duplication of *FLC* may be one mechanism that contributes to the complex transcriptional patterns associated with the perennial cycle. Gene copy number expansion by tandem gene duplication and functional divergence mediated by the accumulation of mutations in *cis* regulatory regions has been proposed to contribute to evolution of complex traits in both plants and mammals [74], [75], [76]. In the Brassicacae acquisition of metal hyperaccumulation in *A. halleri* compared to *A. thaliana* was attributed to the amplification of *HEAVY METAL ATPASE 4* (*HMA4*) [77]. In this case, gene amplification increases the expression level of the metal pump encoded by *HMA4* due both to the higher copy number of the gene and to *cis*-acting changes in promoter sequences. Conceivably the increased copy number of *PEP1* may also be associated with an increased expression level and this might be partially responsible for the stronger repression of flowering found in obligate vernalization requiring *A. alpina* accessions such as Pajares.

## Materials and Methods

### Plant materials and growth conditions

*A. alpina* accessions screened are listed in Table S1. The accession Pajares (Paj) was originally collected in the Cordillera Cantábrica mountain in Spain, selfed for six generations by single seed descent and characterized in [5]. The *pep1-1* mutant was previously characterized in [5]. The accessions Dor, Tot and Wca were selfed by single seed descent for six generations. The accession Cza was selfed for two generations and accession Mug was selfed once. F1s for allelism tests with *pep1-1* and Paj were generated using *pep1-1* and Paj as mother plants.

Flowering time was measured when first flower opened and demonstrated as number of days to flower (from the time seeds were put on soil) or number of leaves at flowering. For all flowering time experiments plants were grown in long days cabinets (16 hours light at 20°C and 8 hours dark at 18°C). Duration of flowering season was measured in long day (16 hours light) controlled environment glasshouse.

### Gene expression analysis

For gene expression studies RNA was extracted from apices and leaves using the RNeasy Plant Mini kit (Qiagen) and purified the DNA-*free* Kit (Applied Biosystems). For expression studies, first strand cDNA was synthesized from 1 µg RNA using different primers depending on the aim of the experiment (Table 2). 5′ RACE was performed using the Invitrogen kit on 2.5 µg RNA according to manufacturer's instructions. PCR products after 5′ RACE were cloned in a pGEM-T easy vector and several clones were sequenced using M13 primers.

**Table 2:** List of primers used for expression studies.

Primers used for cDNA synthesis	Primer sequence (5′ - 3′) used for PCR or qRT
**Experiment: Sequencing ** ***PEP1*** ** cDNAs from accessions (** **Figure 3E** **, ** **Table 1** **, Table S2)**	
Oligo-dT	PEP1_5UTRF1: AACGCTTAGTATCTCAGGCGACPEP1_5UTRF2: CCTTCTCGGAGACAGAAGCCPEP1_3UTRR1: AGTCTCTCAGCCATAGAGAG
**Experiment: ** ***PEP1*** ** expression (** **Figure 2A** **, ** ***PEP1*** ** primers as in ** [**2**] **)**	
Oligo-dT	PEP1(a+b)F: CTTGTCGTCTCCTCCTCTGGPEP1(a+b)R: ACTACGGCGAGAGCAGTTTCAaPP2AF: AGTATCGCTTCTCGCTCCAGAaPP2AR: AACCGGTTGGTCGACTATTG
**Experiment: Expression of different ** ***PEP1*** ** transcripts in accession Dor (** **Figure 5D, 5E, 5F** **)**	
PEP1_3UTRR1AaPP2AR	PEP1(a+b)F: CTTGTCGTCTCCTCCTCTGG [PEP1(a+b)]PEP1(a+b)R: ACTACGGCGAGAGCAGTTTCPEP1aF: GCTTAGTATCTCAGGCGAC [PEP1a]PEP1R: TGCGACGTTTAGAGAAGGTGPEP1bF: GAACAACCGATATTGATGCTC [PEP1b]PEP1R: TGCGACGTTTAGAGAAGGTGAaPP2AF: AGTATCGCTTCTCGCTCCAGAaPP2AR: AACCGGTTGGTCGACTATTG
**Experiment: 5′RACE**	
PEP1(a+b)R	NESTR1: GATCATCTGCATGTCGTTTTCC

### Protein work

To raise a PEP1 antibody the histidine-tagged 127 amino-acid long C-terminal segment of PEP1 was expressed in bacteria and the recombinant protein was purified using the Ni-NTA purification system (Qiagen). Rabbit polyclonal antibody was produced by Eurogentec (Eurogentec, Belgium) using the purified protein as antigen. For western analysis apices of 3 week-old *A. alpina* plants were ground in liquid nitrogen, homogenized in Laemmli buffer and the insoluble material was pelleted by centrifugation. Total protein was quantified by Amido-black and 70 ug of total protein was separated on a denaturing 10% polyacrylamide gel and blotted onto PVDF membrane. Membrane was blocked with 5% milk-TBS for 1 h and incubated overnight with PEP1 polyclonal antibody serum diluted 1∶5000 in 5% milk. Anti-rabbit IgG- (Abcam Ab97064) was used as secondary antibody diluted 1∶5000, and chimioluminiscence was visualized using the LAS4000 imaging system (Fujifilm).

### Cloning and sequencing *PEP1* alleles

The *PEP1* locus from the Paj accession was previously sequenced in [5]. For sequencing the *PEP1* locus from the accession Dor a BAC library was screened and the genomic region spanning exon 1b to exon 7 was sequenced by direct sequencing. A 10 Kb between exon 1a to the exon 1b was separately amplified from genomic DNA using the Roche High Fidelity Taq polymerase, cloned into pGEM-T easy vector and sequenced. Sequences were then aligned to get a Dor *PEP1* consensus sequence. The *PEP1* locus from accession Cza was amplified into 1–2 kb overlapping fragments, cloned into pGEM-T easy vector and sequenced. To obtain the *PEP1* allele from the accession Tot the Tot genome was sequenced using next generation sequencing. Genomic DNA from Tot was extracted using the Maxi kit (Qiagen). Library preparation and next generation sequencing 2x-100 bp was performed in the Max Planck Genome Centre (Cologne, Germany) using Illumina HighSeq 2000 loaded into one lane sequencing flow cell. Primers used for sequencing the *PEP1* alleles are available on request. To screen for the presence of exon1a and exon1b among the accessions, genomic DNA from each accession was amplified using Ex1F which is a common exon1a and exon1b forward primer (5′-CCGTAGCTCTCCTTGTCGTC-3′) with intron 1a specific reverse primer Intr1aR (5′-ACAACATCAAKTATGCATCAC-3′, K: G/T) or intron 1b specific reverse primer Intr1bR (5′-ATTTCCMAGAGGCACCAAAG-3′, M: T/A). PCR conditions used 94°C 1 min; 60°C, 40 sec; 72°C 1.5 min (35 cycles) and 72°C, 5 min.

### Accession numbers

The sequence data presented in this paper have been submitted to GeneBank (http://www.ncbi.nlm.nih.gov/Genbank/) with the following accession numbers: JX310558, JX310559, JX878519 and KC123236-KC123241.

## Supporting Information

Figure S1 Number of leaves at flowering of non-vernalization requiring *A. alpina* accessions under long days (16 hours light) compared to *pep1-1* mutant and the accession Paj.
(PDF)

Figure S2 Number of leaves at flowering of F1 hybrids resulting from crosses of non-vernalization requiring accession with *pep1-1* mutant and Paj. Plants grown in long days without vernalization. The *pep1-1* mutant and Paj were used as controls.
(PDF)

Figure S3 Alignment of MADs box sequences of FLC homologues. Base substitution in Dor PEP1a causes an aminoacid substitution conserved among the FLC homologues.
(PDF)

Table S1 *A. alpina* accessions used in this study.
(PDF)

Table S2 *PEP1* splicing forms in early-flowering *A. alpina* accessions. Multiple cDNAs analysed in Table 1 for the accessions Dor, Tot, Wca, Cza and Mug also contained splicing forms of *PEP1*. The number of clones recovered from each accession in shown in the “Clones” column. *PEP1* in the accession Paj is also differentially spliced but splicing forms are rare compared to the canonical Pajares ORF [5]. The full-length *PEP1* cDNA sequence of the vernalization-requiring accession Paj is used as a reference (row highlighted in grey). Nucleotide polymorphisms compared to Paj *PEP1* cDNA sequence obtained for each accession are presented. Nucleotide (nucl.) position and aminoacid (a.a.) changes compared to Paj are mentioned in rows above the grey row. * indicates sequences containing a stop codon.
(PDF)
