# Supplementary figures and images for: PEP1 of Arabis alpina Is Encoded by Two Overlapping Genes That Contribute to Natural Genetic Variation in Perennial Flowering
Source: PLoS Genet. 2012 Dec 20;8(12):e1003130. doi: 10.1371/journal.pgen.1003130 (PMC3527215; doi:10.1371/journal.pgen.1003130)

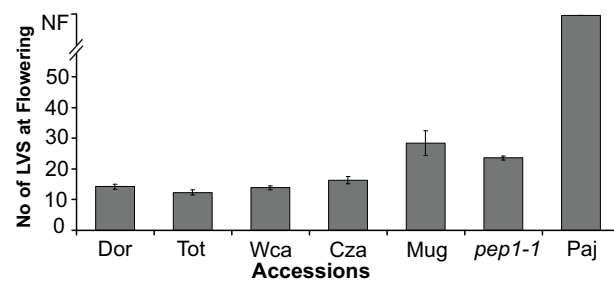

Figure S1

Supplement: Figure S1 — Number of leaves at flowering of non-vernalization requiring A. alpina accessions under long days (16 hours light) compared to pep1-1 mutant and the accession Paj. (PDF) [file pgen.1003130.s001.pdf]

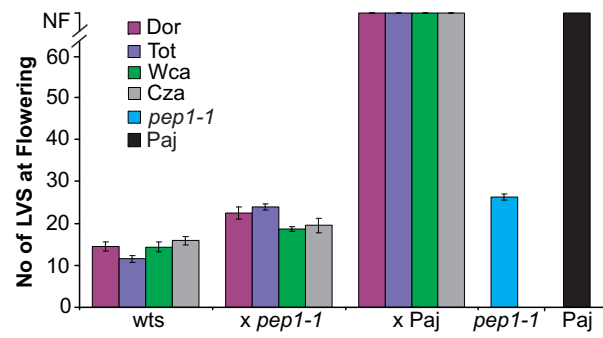

Figure S2

Supplement: Figure S2 — Number of leaves at flowering of F1 hybrids resulting from crosses of non-vernalization requiring accession with pep1-1 mutant and Paj. Plants grown in long days without vernalization. The pep1-1 mutant and Paj were used as controls. (PDF) [file pgen.1003130.s002.pdf]
