# Supplementary material for: PEP1 of Arabis alpina Is Encoded by Two Overlapping Genes That Contribute to Natural Genetic Variation in Perennial Flowering
Source: PLoS Genet. 2012 Dec 20;8(12):e1003130. doi: 10.1371/journal.pgen.1003130 (PMC3527215; doi:10.1371/journal.pgen.1003130)
Supplement: Figure S3 — Alignment of MADs box sequences of FLC homologues. Base substitution in Dor PEP1a causes an aminoacid substitution conserved among the FLC homologues. (PDF) [file pgen.1003130.s003.pdf]

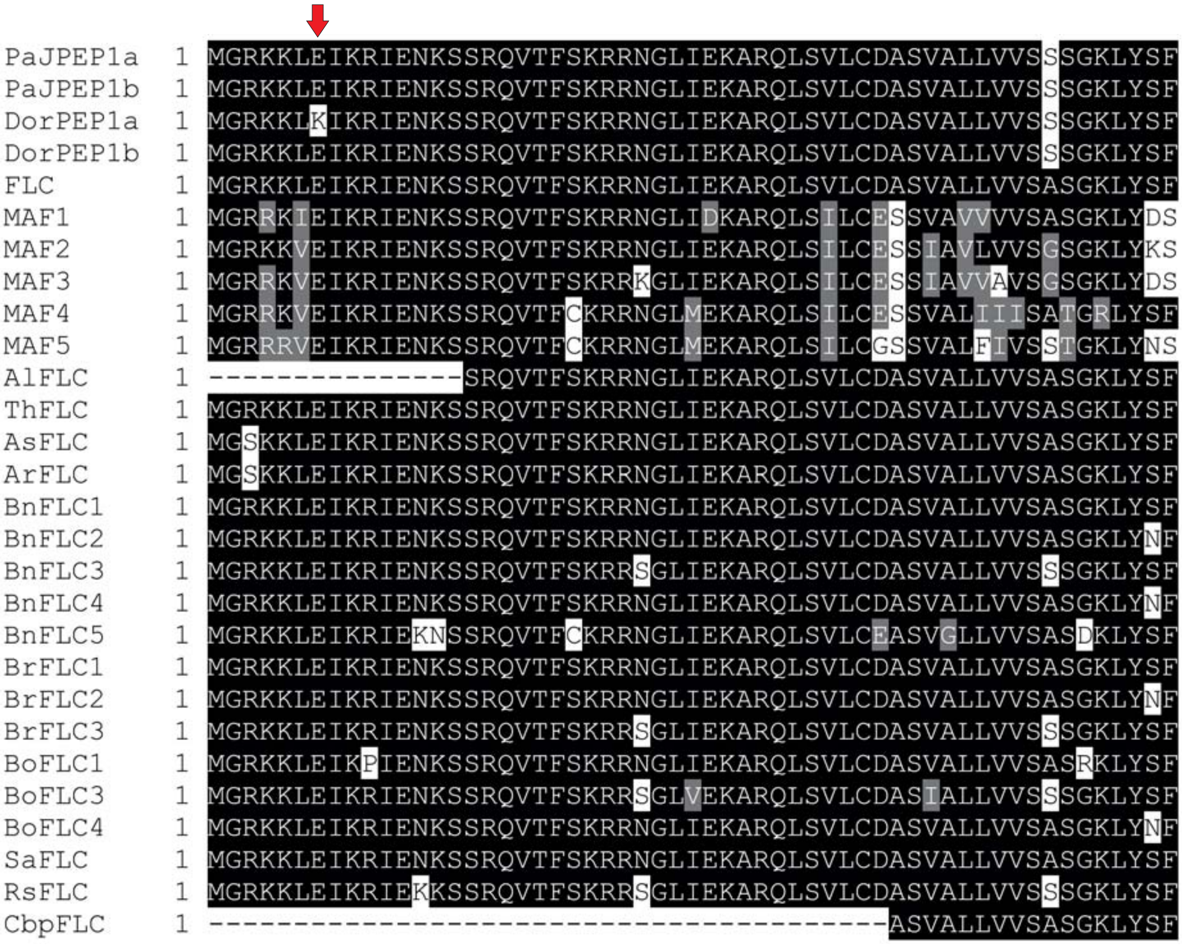
PaJPEP1a 1 MGRKKLEIKRIENKSSRQVTFSKRRNGLIEKARQLSVLCDASVALLVVSSSGKLYSF
PaJPEP1b 1 MGRKKLEIKRIENKSSRQVTFSKRRNGLIEKARQLSVLCDASVALLVVSSSGKLYSF
DorPEP1a 1 MGRKKLKIKRIENKSSRQVTFSKRRNGLIEKARQLSVLCDASVALLVVSSSGKLYSF
DorPEP1b 1 MGRKKLEIKRIENKSSRQVTFSKRRNGLIEKARQLSVLCDASVALLVVSSSGKLYSF
FLC 1 MGRKKLEIKRIENKSSRQVTFSKRRNGLIEKARQLSVLCDASVALLVVSASGKLYSF
MAF1 1 MGRRKIEIKRIENKSSRQVTFSKRRNGLIDKARQLSILCESSVAVVVVSASGKLYDS
MAF2 1 MGRKKVEIKRIENKSSRQVTFSKRRNGLIEKARQLSILCESSIAVLVVSGSGKLYKS
MAF3 1 MGRRKVEIKRIENKSSRQVTFSKRRKGLIEKARQLSILCESSIAVVAVSGSGKLYDS
MAF4 1 MGRRKVEIKRIENKSSRQVTFCKRRNGLMEKARQLSILCESSVALIIISATGRLYSF
MAF5 1 MGRRRVEIKRIENKSSRQVTFCKRRNGLMEKARQLSILCGSSVALFIVSSTGKLYNS
AlFLC 1 ---------------SRQVTFSKRRNGLIEKARQLSVLCDASVALLVVSASGKLYSF
ThFLC 1 MGRKKLEIKRIENKSSRQVTFSKRRNGLIEKARQLSVLCDASVALLVVSASGKLYSF
AsFLC 1 MGSKKLEIKRIENKSSRQVTFSKRRNGLIEKARQLSVLCDASVALLVVSASGKLYSF
ArFLC 1 MGSKKLEIKRIENKSSRQVTFSKRRNGLIEKARQLSVLCDASVALLVVSASGKLYSF
BnFLC1 1 MGRKKLEIKRIENKSSRQVTFSKRRNGLIEKARQLSVLCDASVALLVVSASGKLYSF
BnFLC2 1 MGRKKLEIKRIENKSSRQVTFSKRRNGLIEKARQLSVLCDASVALLVVSASGKLYNF
BnFLC3 1 MGRKKLEIKRIENKSSRQVTFSKRRSGLIEKARQLSVLCDASVALLVVSSSGKLYSF
BnFLC4 1 MGRKKLEIKRIENKSSRQVTFSKRRNGLIEKARQLSVLCDASVALLVVSASGKLYNF
BnFLC5 1 MGRKKLEIKRIEKNSSRQVTFCKRRNGLIEKARQLSVLCEASVGLLVVSASDKLYSF
BrFLC1 1 MGRKKLEIKRIENKSSRQVTFSKRRNGLIEKARQLSVLCDASVALLVVSASGKLYSF
BrFLC2 1 MGRKKLEIKRIENKSSRQVTFSKRRNGLIEKARQLSVLCDASVALLVVSASGKLYNF
BrFLC3 1 MGRKKLEIKRIENKSSRQVTFSKRRSGLIEKARQLSVLCDASVALLVVSSSGKLYSF
BoFLC1 1 MGRKKLEIKPIENKSSRQVTFSKRRNGLIEKARQLSVLCDASVALLVVSASRKLYSF
BoFLC3 1 MGRKKLEIKRIENKSSRQVTFSKRRSGLVEKARQLSVLCDASIALLVVSSSGKLYSF
BoFLC4 1 MGRKKLEIKRIENKSSRQVTFSKRRNGLIEKARQLSVLCDASVALLVVSASGKLYNF
SaFLC 1 MGRKKLEIKRIENKSSRQVTFSKRRNGLIEKARQLSVLCDASVALLVVSASGKLYSF
RsFLC 1 MGRKKLEIKRIEKKSSRQVTFSKRRSGLIEKARQLSVLCDASVALLVVSSSGKLYSF
CbpFLC 1 --ASVALLVVSASGKLYSF

Figure S3
