# Supplementary material for: PEP1 of Arabis alpina Is Encoded by Two Overlapping Genes That Contribute to Natural Genetic Variation in Perennial Flowering
Source: PLoS Genet. 2012 Dec 20;8(12):e1003130. doi: 10.1371/journal.pgen.1003130 (PMC3527215; doi:10.1371/journal.pgen.1003130)
Supplement: Table S1 — A. alpina accessions used in this study. (PDF) [file pgen.1003130.s004.pdf]

Table S1. *A. alpina* accessions used in this study

Accession	Abbreviation	Location	Provided/collected by
Pajares	Paj	Cordillera Cantabrica mountain, Spain	Carlos Alonso-Blanco
Jaca	Jac	Jaca, Pyrenees, Spain	Carlos Alonso-Blanco
Val d' Aram Artieda	Ara	Val d' Aram Artieda, Pyrenees, Spain	Martin Lysak
Col du Perthus	Per	Col du Perthus, Pyrenees, France	Martin Lysak
Benasque	Ben	Benasque, Pyrenees, Spain	Martin Lysak
Apuan-158	Apu158	Apuan Alps, Italy	Steve Ansell
Apuan-166	Apu166	Apuan Alps, Italy	Steve Ansell
Val d'Arcia	Var	Monte Pelmo, Italy	Genbank Osnabrück (95-0357-10-04)
Monte Baldo	Bal	Monte Baldo, Italy	Genbank Osnabrück (96-0083-10-00)
Maritime-160	Mar160	Maritime Alps, Italy	Steve Ansell
Maritime-161	Mar161	Maritime Alps, Italy	Steve Ansell
South Tyrol	Sty	South Tyrol, Italy	Frank Eikelmann
Chamechaude	Cha	Chartreuse, France	Jörg Wunder
Wilderswil	Wil	Berner Oberland, Switzerland	Genbank Osnabrück (07-0025-10-00)
Totes Gebirge	Tot	Totes Gebirge mountain range, Austria, 1600m	Frank Eikelmann
Dorfertal	Dor	Dorfertal valley, National Park Hohe Tauern, East Tyrol, Austria, 1650m	Bonn botanic gardens
Czarna Gòra	Cza	Pieniny Mountains, Poland (N 49.428333°, E 20.1241667°), 600 m	Genbank Osnabrück (08-0002-10-00)
West Carpathians	Wca	West Carpathians	Marburg botanic gardens (SK-0-DR-016546)
Bratislava	Bra	Bratislava, Czech Republic	Genbank Osnabrück (92-25-0031-10)
Bavarian Alps 1	Bav1	Bavarian Alps, Germany, 1950 m	Frank Eikelmann
Bavarian Alps 2	Bav2	Bavarian Alps, Germany, 2730 m	Frank Eikelmann
Muggendof	Mug	Frankische Schweiz, Germany (N 49.79073°, E 11.27414°), 325m	Jörg Wunder
Klumpertal	Klu	Frankische Schweiz, Germany (N 49.74240°, E 11.42693°), 400m	Jörg Wunder
Finnmark	Fin	Finnmark, Norway	Genbank Osnabrück (94-0225-10-00)
