# Supplementary material for: PEP1 of Arabis alpina Is Encoded by Two Overlapping Genes That Contribute to Natural Genetic Variation in Perennial Flowering
Source: PLoS Genet. 2012 Dec 20;8(12):e1003130. doi: 10.1371/journal.pgen.1003130 (PMC3527215; doi:10.1371/journal.pgen.1003130)
Supplement: Table S2 — PEP1 splicing forms in early-flowering A. alpina accessions. Multiple cDNAs analysed in Table 1 for the accessions Dor, Tot, Wca, Cza and Mug also contained splicing forms of PEP1. The number of clones recovered from each accession in shown in the “Clones” column. PEP1 in the accession Paj is also differentially spliced but splicing forms are rare compared to the canonical Pajares ORF [5]. The full-length PEP1 cDNA sequence of the vernalization-requiring accession Paj is used as a reference (row highlighted in grey). Nucleotide polymorphisms compared to Paj PEP1 cDNA sequence obtained for each accession are presented. Nucleotide (nucl.) position and aminoacid (a.a.) changes compared to Paj are mentioned in rows above the grey row. * indicates sequences containing a stop codon. (PDF) [file pgen.1003130.s005.pdf]

Table S2. ***PEP1*** **splicing forms in early-flowering** ***A. alpina*** **accessions**

	Clones	Exon 1	Exon 3		Exon 4			Exon 5	Exon 6	Retained introns
**nucl.**		100	325-333		415-467	433	411	513-517	556-597	
**a.a.**		6<br>E/K	82<br>D/S	83-85<br>-3a.a.	100-139<br>-40a.a.	118<br>V/I		*	159-172<br>-14a.a.	
**Paj**		G				G	C			
**Dor**	1	A	-9							
	3	A							-42	
	1	A								2
**Tot**	8					A	T		-42	
	1					A	T	-5	-42	
	1					A	T		-42	2
	3					A	T			6
**Wca**	5								-42	
	1				-52				-42	
